# Circulating Calreticulin Is Increased in Myelofibrosis: Correlation with Interleukin-6 Plasma Levels, Bone Marrow Fibrosis, and Splenomegaly

**DOI:** 10.1155/2016/5860657

**Published:** 2016-09-08

**Authors:** Daria Sollazzo, Dorian Forte, Nicola Polverelli, Margherita Perricone, Marco Romano, Simona Luatti, Nicola Vianelli, Michele Cavo, Francesca Palandri, Lucia Catani

**Affiliations:** Department of Experimental, Diagnostic and Specialty Medicine, Institute of Hematology “L. and A. Seràgnoli”, University of Bologna, Bologna, Italy

## Abstract

Myelofibrosis (MF) is a clonal neoplasia of the hemopoietic stem/progenitor cells associated with genetic mutations in the Janus kinase 2 (*JAK2*), myeloproliferative leukemia virus oncogene (*MPL*), and calreticulin (*CALR*) genes. MF is also characterized by a state of chronic inflammation. Calreticulin (CRT), as a multifunctional protein, is involved in a spectrum of cellular processes including inflammation, autoimmunity, and cancer initiation/progression. Based on this background, we hypothesised that in MF circulating CRT might reflect the inflammatory process. In the present study we show that circulating CRT is increased in MF patients compared to healthy controls. Also, in MF, CRT levels highly correlate with bone marrow fibrosis, splenomegaly, and Interleukin-6 (IL-6) plasma levels. In turn, higher IL-6 levels also correlated with disease severity in terms of increased spleen size, bone marrow fibrosis, number of circulating CD34^+^ cells, and lower hemoglobin values. These results demonstrate that the circulating CRT takes part in the inflammatory network of MF and correlates with aggressiveness of the disease.

## 1. Introduction

Myelofibrosis (MF) is a Philadelphia-negative myeloproliferative neoplasm (Ph-neg MPN) that may arise de novo (Primary Myelofibrosis, PMF) or after Essential Thrombocythemia (ET; PET-MF) and Polycythemia Vera (PV; PPV-MF). MF is a rare blood cancer with an incidence of about 0.58 new cases per 100.000 people per year but with higher prevalence because of a chronic and disabling course leading always to death due to progression and disease-related or treatment-related complications. It is a clonal disorder of the hemopoietic stem/progenitor cells which is clinically characterized by worsening constitutional symptoms, progressive splenomegaly, bone marrow (BM) fibrosis, and cytopenias as well as by an increased risk to develop thrombotic complications and acute leukemia [1–3].

Driver mutations in Janus kinase 2 (*JAK2*), calreticulin (*CALR*), and myeloproliferative leukemia virus oncogene (*MPL*) have been reported. However, less than 10% of patients have nonmutated* JAK2*,* MPL*, and* CALR* genes (“triple-negative”). Regardless of molecular status, all patients have dysregulation in the JAK/STAT signaling [2–6].

Together with molecular abnormalities, MF is characterized by abnormal expression of several proinflammatory and immunoregulating cytokines secreted by activated leukocytes and platelets/megakaryocytes. This inflammatory microenvironment has emerged as a key player in MF pathogenesis [7–12].

Physiologically, CRT was first described as an endoplasmic reticulum protein responsible for Ca^2+^ homeostasis and glycoprotein folding; currently, CRT is recognized as a multifunctional chaperone detected in other cellular compartments, as well as extracellularly, where it is involved in cell proliferation, phagocytosis, apoptosis, adhesion, and innate and adaptive immune processes including cancer cell elimination by immunogenic cell death and fibrosis [13]. CRT overexpression is linked to various pathological conditions including chronic inflammatory diseases, autoimmunity, fibrosis-related disorders, and malignant evolution [14–18]. In MF, the mutated CRT protein was found to constitutively activate the MPL receptor signaling [19, 20].

Given the CRT involvement in inflammation, fibrosis, and cancer, we hypothesised that in MF circulating CRT might reflect the inflammatory process. Here, we characterized the circulating CRT levels of MF patients. Moreover, we investigated the correlation between CRT levels and various clinical and laboratory parameters.

## 2. Materials and Methods

### 2.1. Study Population

Peripheral blood (PB) was obtained from 30 patients with MF in chronic phase and from 10 healthy age-matched volunteers. The diagnosis of MF was made according to WHO 2008 criteria [21]. Patients and controls provided written informed consent for the study. This study was approved by the medical Ethical Committee of the University Hospital of Bologna and was conducted in accordance with the Declaration of Helsinki.

### 2.2. Assay of Circulating Proteins

Here we analyzed the plasma levels of CRT in patients/controls. EDTA-anticoagulated PB was centrifuged for 15 minutes at 1000 ×g within 30 minutes of collection. The plasma was then collected and stored at −80°C until quantification. CRT was evaluated by a commercially available ELISA assay (Cusabio Biotech Co., Wuhan, China), according to the manufacturer's instructions. Briefly, a standard curve of 100 *μ*L aliquots of known concentrations of recombinant CRT was run and triplicate 100 *μ*L samples were added to the wells. CRT binding was detected using a biotin/avidin system. The plates were then assessed by ELISA on a plate reader at 450 nm. The Ciraplex™ immunoassay kit/Human 9-Plex Array (Aushon BioSystems, Billerica, MA, USA) was used for the measurement of circulating IL-6 and TNF-*α*.

### 2.3. Molecular Pattern

Molecular analyses were assessed at diagnosis or before treatment's start on DNA obtained from granulocytes. Driver mutations were analyzed as previously described [22]. Specifically, *JAK*2^V617F^ mutation was evaluated with ipsogen* JAK2* MutaQuant Kit. The percentage of mutant *JAK*2^V617F^ allele was expressed as the ratio of *JAK*2^V617F^ copies to total copy number (CN) of* JAK2* (CN of *JAK*2^V617F^ + CN of* JAK2* wild type).* CALR* exon 9 sequencing was performed by Next Generation Sequencing (NGS) approach with GS Junior (Roche-454 platform); analysis was carried out with AVA Software (GRCh38 as referenced). Rare* CALR* mutations identified by NGS were confirmed by Sanger sequencing.* MPL* mutations were investigated by ipsogen* MPL W515K/L* MutaScreen Kit and by Sanger sequencing (for* MPLS505N* and other secondary exon 10 mutations).

### 2.4. Cytogenetic Analysis

Chromosome banding analysis was performed on BM cells by standard banding techniques according to the International System for Human Cytogenetic Nomenclature [23]. At least 20 metaphases were required. Unfavorable karyotype was defined according to the Dynamic International Prognostic Score System (DIPSS) plus [24] and included complex karyotype or single or two abnormalities including +8, −7/7q-, i(17q), −5%5q-, 12p-, inv(3), or 11q23 rearrangement.

### 2.5. Statistical Analysis

Statistical analyses (Wilcoxon test and Spearman correlation analysis) were performed using GraphPad (GraphPad Software Inc., La Jolla, CA). All *p* values were considered statistically significant when *p* ≤ 0.05 (two-tailed).

## 3. Results 

A total of 30 MF patients were investigated: *JAK*2^*V*617*F*^- (16 cases),* CALR*- (10 cases), and* MPL*- (3 cases) mutated. One patient was triple-negative.* CALR*-mutated patients were type 1 (8 cases) and type 2 (2 cases). Patients characteristics are shown in Table 1. Fifteen patients were at diagnosis. Thirteen patients received previous therapies for MF (hydroxyurea (10 cases) and ruxolitinib (3 cases)); however, at the time of the study, they were untreated for at least 2 months.

**Figure 1:**
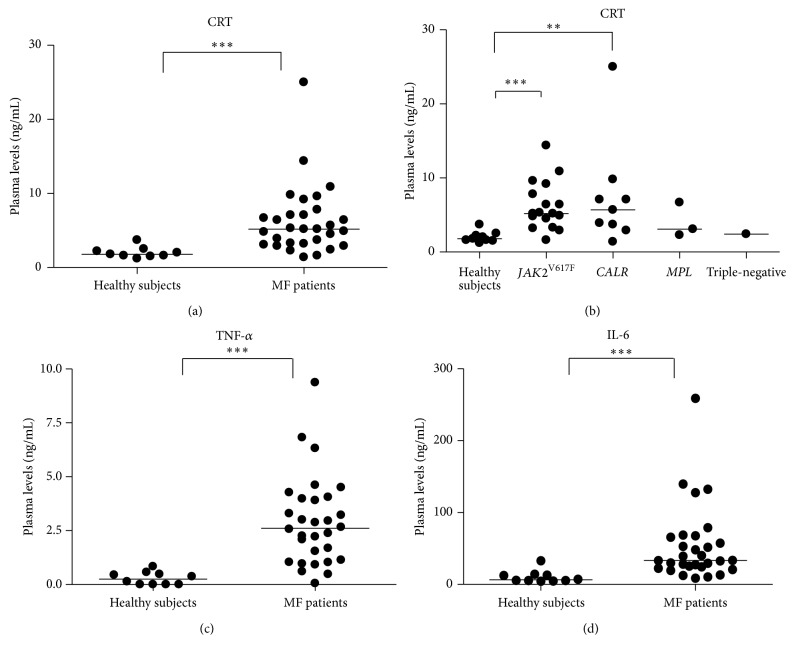
*Analysis of the circulating levels of CRT, IL-6, and TNF-α proteins*. The CRT plasma levels of total MF (a) or MF subdivided into* JAK2* ^*V617F*^-mutated (*n* = 16),* CALR*-mutated (*n* = 10),* MPL*-mutated (*n* = 3), and triple-negative-mutated (*n* = 1) groups (b) were measured by ELISA. Compared with age-matched controls (HD; *n* = 10), CRT plasma levels were significantly increased in MF patients (*p* = 0.0028). Of note, there was no significant difference between the mutated groups. Irrespective of mutational status, TNF-*α* (c) and IL-6 (d) blood plasma levels were also increased in MF (*p* = 0.008). For all graphs, one symbol represents one individual, and the height of the bar represents the median value. ^*∗∗*^ *p* ≤ 0.01; ^*∗∗∗*^ *p* ≤ 0.001.

As shown in Figure 1(a), we found significantly higher CRT plasma levels in MF patients as compared with healthy subjects (median, 5.2 ng/mL, and range, 1.4–25, versus median, 1.8 ng/mL, and range, 1.2–3.7; *p* = 0.0028). Comparing CRT plasma levels of *JAK*2^*V*617*F*^ and* CALR*-mutated patients, no significant differences were observed (Figure 1(b)). Even though few patients were studied, CRT plasma levels of MPL-mutated and triple-negative patients were superimposable to the other MF patients (Figure 1(b)). CRT plasma levels of patients at diagnosis were not significantly different from those of the other patients. No correlation was observed between circulating CRT levels and hemoglobin levels, white blood cells/platelets count, and circulating CD34^+^ cells number.

**Figure 2:**
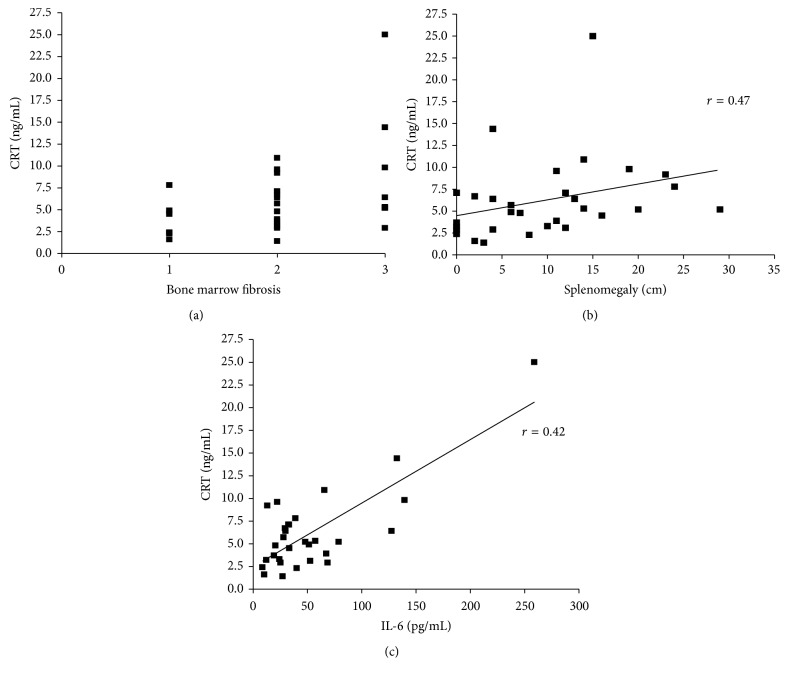
*Correlation between CRT plasma levels and BM fibrosis, splenomegaly, and circulating IL-6*. Circulating CRT positively correlates with fibrosis, splenomegaly, and soluble IL-6 in MF. Scatterplots demonstrating correlation between the plasma levels of CRT and BM fibrosis (*p* = 0.038; *r* = 0.39), splenomegaly (*p* = 0.0089; *r* = 0.47), and circulating IL-6 (*p* = 0.028; *r* = 0.42) in MF patients (a, b, and c, resp.) are shown. *x*-axis of (a) shows BM fibrosis scale.

**Figure 3:**
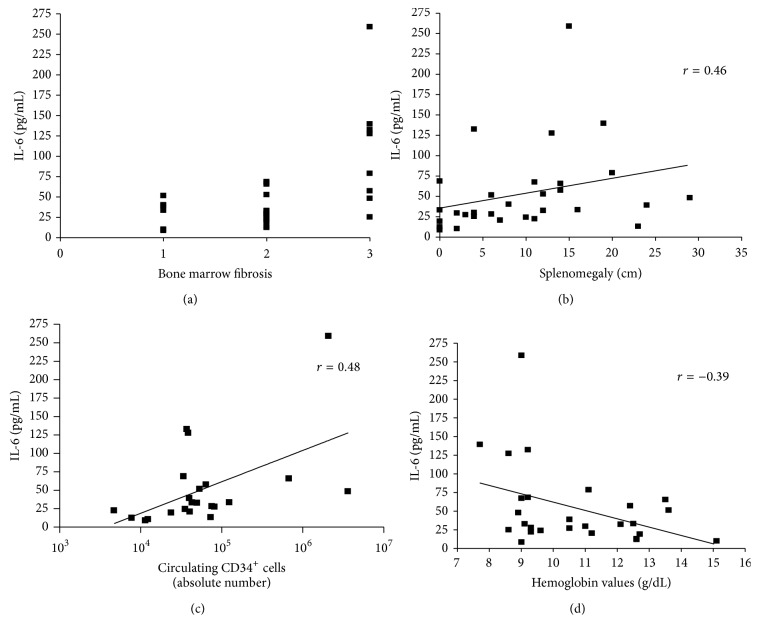
*Correlation between IL-6 plasma levels and BM fibrosis, splenomegaly, number of circulating CD34* ^*+*^ *cells, and hemoglobin values*. Irrespective of mutational status, IL-6 plasma levels correlated with BM fibrosis (*p* = 0.0056; *r* = 0.49), splenomegaly (*p* = 0.018; *r* = 0.46), and the absolute number of circulating CD34^+^ cells (*p* = 0.029; *r* = 0.48) and negatively correlated with hemoglobin values (*p* = 0.047; *r* = −0.39); (a, b, c, and d, resp.). *x*-axis of (a) shows BM fibrosis scale.

Along with CRT plasma levels, circulating TNF-*α* (median: 2.62 pg/mL; range: 0.05–9.37) and IL-6 (median: 33.3 pg/mL; range: 8.7–258.9) were also increased in MF patients as compared to healthy subjects (median, 0.26 pg/mL, and range, 0–0.84, and median, 6.37 pg/mL, and range, 4.5–32.8, resp.; *p* = 0.008) (Figures 1(c) and 1(d)). TNF-*α* and IL-6 plasma levels were not affected by mutational status and allele burden (data not shown). Interestingly, in MF, irrespective of patients being at diagnosis or not, there was a positive correlation between the plasma levels of CRT and BM fibrosis (*p* = 0.038; *r* = 0.39), splenomegaly (*p* = 0.0089; *r* = 0.47), and circulating IL-6 (*p* = 0.028; *r* = 0.42) (Figures 2(a), 2(b), and 2(c)). This correlation was also irrespective of mutational status (comparing *JAK*2^*V*617*F*^-mutated and* CALR*-mutated patients). In turn, IL-6 plasma levels correlated with BM fibrosis (*p* = 0.0056; *r* = 0.49), splenomegaly (*p* = 0.018; *r* = 0.46), and the number of circulating CD34^+^ cells (*p* = 0.029; *r* = 0.48) and correlated negatively with hemoglobin values (*p* = 0.047; *r* = −0.39; Figures 3(a), 3(b), 3(c), and 3(d)).

## 4. Discussion

There has been a lack of understanding regarding the role of soluble CRT in MF. The first result of the study is that in MF CRT plasma levels are increased compared to healthy controls. CRT has been found to have a preferential expression in megakaryocyte/platelets either from normal subjects or from patients with Ph-neg MPN (and regardless of mutation status) [25]. Therefore, these cells, which show abnormal number/function in MF, are likely to be the major contributors to the augmented amount of circulating CRT. Previous studies support the hypothesis that extracellular and soluble CRT is mainly released from dead, dying, or inflamed/stressed cells [13–16]. Consequently, the high CRT levels detected in MF may primarily be due to the chronic inflammatory state that characterizes both the marrow and peripheral niches and reflect impairment in tissue homeostasis.

The second result is that CRT plasma levels are equally increased in JAK2^V617F^-mutated and CALR-mutated MF patients. In this study, we used an antibody that is directed against the N terminus of CRT and is expected to label both mutated and unmutated proteins. Therefore, the circulating protein that was detected in* CALR*-positive patients is likely to be the sum of mutated (hemopoietic restricted) and unmutated molecules. This datum may suggest that the acquisition of mutations in the* CALR* gene, although causing the hyperactivation of the MPL receptor [19, 20], does not induce an increased circulating CRT amount.

Herein, we therefore demonstrated that CRT protein levels were found to directly correlate with the clinical aggressiveness of the disease in terms of larger spleen size and more severe marrow fibrosis. In addition, we found a direct correlation between circulating plasma levels of CRT and IL-6, one of the most potent proinflammatory cytokines which is upregulated in MF [26]. In turn, higher IL-6 levels also correlated with disease severity in terms of increased spleen size, marrow fibrosis, number of circulating CD34^+^ cells, and lower hemoglobin values. Even though these correlations were weak (*r* always below 0.5), all together point out the involvement of CRT in the inflammatory network and in disease aggressiveness. The correspondence between CRT and IL-6 plasma levels may be at least partially justified by the recent discovery that soluble CRT induces active mRNA transcription through MAPK and NF-kB activation in macrophages, thereby augmenting their IL-6 and TNF-*α* production [27]. In addition, recently, conditioned media from cells expressing type I mutant* CALR* have been shown to exaggerate cytokine production from normal monocytes [28]. It is therefore likely that in MF the increased circulating CRT may contribute to the disease-related inflammation/fibrosis through positively enhancing IL-6 production. By contrast, despite the fact that TNF-*α* is a negative regulator of CRT expression [29], no correlation was observed between circulating CRT and TNF-*α* plasma levels in our MF patients, suggesting the presence of a TNF-*α*-independent mechanism of regulation.

Taken together, our data highlight the role of this protein in the inflammatory network of MF. A mutual interaction among CRT and other inflammatory cytokines including IL-6 may indeed contribute to the generation/maintenance of inflammation/fibrosis of MF.

Potential limitation of the present study is the small sample size of patients. Nonetheless, our data create the rational basis for future studies investigating the role of circulating CRT in the inflammatory network of MF and other Ph-negative MPNs in larger cohorts of patients. Notably, due to correlation with fibrosis and splenomegaly, circulating CRT measurement may be useful in clinical practice.

## 5. Conclusion

We conclude that the elevated plasma CRT levels in MF patients parallel the degree of disease activity and inflammatory state. This may identify patients with more severe disease who might benefit from tailored therapy.

## Figures and Tables

**Table 1:** Patients characteristics according to mutational status. Compared to CALR-mutated patients, patients with JAK2^V617F^ mutation were older (*p* = 0.01) and had higher hemoglobin levels (*p* = 0.04).

Characteristics	JAK2^V617F^-mutated patients (number = 16)	CALR-mutated patients (number = 10)	MPL-mutated patients (number = 3)	“Triple-negative” patients (number = 1)
Median age, years (range)	73 (67–84)	65.5 (44–82)	76 (72–76)	67
Male sex, number (%)	9 (56)	6 (60)	1 (33.3)	0 (0)
Median allele burden, % (range)	89 (0.4–99)	56.5 (52–98)	NA	NA
Median WBC, ×10^9^/L (range)	10.6 (2.5–157.6)	7.15 (2.3–48.3)	6.2 (4.9–25)	6.7
Median hemoglobin, g/dL (range)	11.1 (8.6–15.1)	9.3 (7.7–14)	9.7 (7.2–9.9)	8.5
Median platelet count, ×10^9^/L (range)	280 (41–507)	195.5 (86–419)	196 (46–303)	632
High/intermediate 2 IPSS category, number (%)	9 (56)	6 (60)	3 (100)	1 (100)
Unfavorable karyotype, number (%)	8 (50)	2 (20)	2 (66.6)	1 (100)
Diagnosis, number (%)				
PMF	8 (50)	6 (60)	3 (100)	1 (100)
PET	3 (19)	2 (20)	—	—
PPV	5 (31)	2 (20)	—	—
BM fibrosis grade ≥ 2, number (%)	12 (75)	10 (100)	2 (66.6)	0 (0)
Patients with splenomegaly, number (%)	14 (87.5)	8 (80)	3 (100)	0 (0)

WBC: white blood cells; IPSS: International Prognostic Scoring System; PMF: primary myelofibrosis; NA: not available. Splenomegaly was evaluated by palpation as cm below costal margin. Only patients with a spleen palpable ≥ 5 cm below costal margin by palpation were considered as carrying splenomegaly.
